# Pharmacology of ginsenosides: a literature review

**DOI:** 10.1186/1749-8546-5-20

**Published:** 2010-06-11

**Authors:** Kar Wah Leung, Alice Sze-Tsai Wong

**Affiliations:** 1 Department of Biology, The Hong Kong University of Science and Technology, Clear Water Bay, Hong Kong SAR, PR China; 2 School of Biological Sciences, University of Hong Kong, Pokfulam Road, Hong Kong SAR, PR China

## Abstract

The therapeutic potential of ginseng has been studied extensively, and ginsenosides, the active components of ginseng, are shown to be involved in modulating multiple physiological activities. This article will review the structure, systemic transformation and bioavailability of ginsenosides before illustration on how these molecules exert their functions via interactions with steroidal receptors. The multiple biological actions make ginsenosides as important resources for developing new modalities. Yet, low bioavailability of ginsenoside is one of the major hurdles needs to be overcome to advance its use in clinical settings.

## Review

### Background

*Panax ginseng *(*Renshen*, Chinese ginseng) is commonly used either by itself or in combination with other medicinal ingredients as a key herb in Chinese medicine. A member of the Araliaceae family, the genus name Panax was derived from the Greek word meaning "all-healing" first coined by the Russian botanist Carl A. Meyer. The Panax family consists of at least nine species, including *P. ginseng*, *Panax quinquefolium *(*Xiyangshen*, American ginseng), *Panax notoginseng *(*Sanqi*) and *Panax japonicus *(Japanese ginseng). The worldwide sale of ginseng products has estimated to reach US$ 300 million in 2001 [1,2].

Ginseng modulates blood pressure, metabolism and immune functions [3-6]. The action mechanism of ginseng had not been known until ginsenosides were isolated in 1963 [7,8]. Much effort has since been focused on evaluating the function and elucidating the molecular mechanism of each ginsenoside. Number of publications on ginseng and ginsenosides has been growing exponentially since 1975 according to the Pubmed entry.

### Ginsenosides are the pharmacologically active components in ginseng

Ginsenosides are triterpene saponins. Most ginsenosides are composed of a dammarane skeleton (17 carbons in a four-ring structure) with various sugar moieties (e.g. glucose, rhamnose, xylose and arabinose) attached to the C-3 and C-20 positions [9,10]. Ginsenosides are named as 'Rx', where the 'R' stands for the root and the 'x' describes the chromatographic polarity in an alphabetical order [7], for example, Ra is the least polar compound and Rb is more polar than Ra. Over 30 ginsenosides have been identified and classified into two categories: (1) the 20(S)-protopanaxadiol (PPD) (Rb1, Rb2, Rb3, Rc, Rd, Rg3, Rh2, Rs1) and (2) the 20(S)-protopanaxatriol (PPT) (Re, Rf, Rg1, Rg2, Rh1). The difference between PPTs and PPDs is the presence of carboxyl group at the C-6 position in PPDs [9,10]. Moreover, several rare ginsenosides, such as the ocotillol saponin F11 (24-R-pseudoginsenoside) [11] and the pentacyclic oleanane saponin Ro (3,28-O-bisdesmoside) [12] have also been identified.

The quality and composition of ginsenosides in the ginseng plants are influenced by a range of factors bhsuch as the species, age, part of the plant, cultivation method, harvesting season and preservation method [13,14]. For example, ginsenoside Rf is unique to Asian ginseng while F11 is found exclusively in American ginseng. Thus the Rf/F11 ratio is used as a phytochemical marker to distinguish American ginseng from Asian ginseng [15,16]. The overall saponin content in ginseng is directly proportional to its age, reaching a peak level at around 6 years of age [17,18]. Most harvested ginseng roots are air-dried while some are steamed at 100°C for two to four hours before drying, which gives the ginseng a darker appearance known as red ginseng. The red ginseng has a unique saponin profile, with emerging ginsenosides Ra1, Ra2, Ra3, Rf2, Rg4, Rg5, Rg6, Rk1, Rs1 and Rs2 being likely the results of heat transformation and deglycosylation of naturally occurring ginsenosides [19-24]. The presence of these compounds may confirm the folk knowledge that red ginseng is of higher medicinal values than the white one [25].

Sun ginseng is a new type of processed ginseng that is steamed at 120°C. The new process aimed to increase the levels of anti-tumor ginsenosides Rg3, Rg5 and Rk1 [26-30]. Moreover, the butanol-soluble fraction of Sun ginseng is formulated into KG-135 which contains Rk3 Rs3, Rs4, Rs5, Rs6 and Rs7 in addition to the major anti-tumor ginsenosides [31].

### Standardized ginseng extracts

To avoid variability among preparations, many researchers use commercially available standardized ginseng extracts. Two commonly used standardized extracts are G115 from *P. ginseng *(total ginsenoside adjusted to 4%) (Pharmaton SA, Switzerland) and NAGE from *P. quinquefolius *(total ginsenoside content adjusted to 10%) (Canadian Phytopharmaceuticals Corporation, Canada). Studies on these two ginseng extracts using high-performance liquid chromatography (HPLC) found ginsenosides Rb1, Rb2, Rc, Rd, Re and Rg1 in both G115 and NAGE, and ginsenoside Rg2 in G115 only. To compare between G115 and NAGE, G115 has higher Rg1, but NAGE has higher in Rb1 and Re [32-34].

### Ginsenosides are part of the defense mechanisms in ginseng

Similar to plants that produce insect repellents and anti-microbial substances as part of their defense mechanisms, e.g. nicotine from tobacco leaves [35], rotenone from derris tree roots [36], pyrethroids from chrysanthemum flowers [37], and triterpenoids from neem tress [38], evidence suggests that ginsenosides may protect ginseng. Addition of methyl jasmonate (a plant-specific signaling molecule expressed during insect and pathogenic attacks) into ginseng *in vitro *cultures enhances ginsenoside production [39-41]. Naturally occurring ginsenosides are antimicrobial and antifungal; the bitter taste of ginsenosides makes them antifeedant [42-46].

Furthermore, ginsenosides may act as ecdysteroids, the insect molting and metamorphosis hormones, due to the structural similarities between the two groups of chemicals. The ecdysteroids have a steroid backbone with a C-20 sugar side-chain and a C-3 hydroxyl group [47] resembling the structure of most of the PPT-type ginsenosides such as Rg1 and several metabolites of PPDs such as compound Y and compound K. Ecdysteroids differ from ginsenosides in the C-6 position which is occupied by an oxygen group is in the former and a hydrogen or hydroxyl group in the latter [47]. Such difference, however, has minor and non-significant influence on ecdysteroid receptor binding affinity as demonstrated by biochemical analysis [47,48]. The structural similarity suggests that certain naturally occurring ginsenosides may disrupt insects' life cycle by binding to ecdysteroid receptor.

### Biotransformation of ginsenosides

Treatment of various cultured cells by ginsenosides revealed multiple bioactivities, including neuroprotection [49-53], antioxidation [54-56], angiogenesis modulation [57-59] and cytotoxicity [60-62]. However, biotransformation may be required before ginsenosides becoming active in mammalian systems. Recent studies demonstrated that ginsenoside metabolites had greater biological effects than ginsenosides [63-65]. Anti-tumor activities of Rh2 and PD, which are the metabolites of Rg3, are more potent than those of ginsenoside Rg3 [64]. Ginsenosides Rb1, Rb2, Rg1 and Re do not possess the same human liver enzyme cytochrome P450 inhibitory effects of compound K, PT and PD which are the intestinal metabolites of PPTs and PPDs [65].

**Figure 1:**
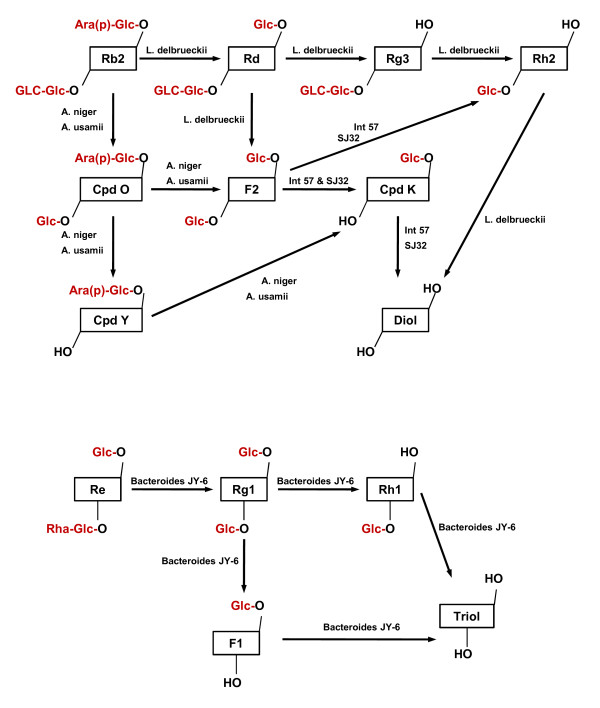
**Biodegradation of ginsenosides by intestinal microflora**. PPDs and PPTs are deglycosylated to end-metabolites protopanaxadiol (PPD) and protopanaxatriol (PPT) respectively. Glc = beta-D-glucopyranosyl; Ara(p) = alpha-L-arabinopyranosyl; Ara(f) = alpha-D-arabinofuranosyl; Rha = alpha-L-rhamnopyranosyl [73-76]

Major ginsenosides, such as Rg1, Rg3, Rb1, Re and Rc, are treated as antigens by mammalian systems. Antibodies against these ginsenosides have been purified from immunized animals [66-70]. Due to their bulky molecular structures, the ginsenosides are poorly membrane permeable and prone to degradation. Oral consumption of ginseng preparations exposes ginsenosides to acid hydrolysis accompanied by side-reactions, glycosyl elimination and epimerization of C-20 sugar moiety [71,72]. The C-3 or C-20 oligosaccharides are also cleaved by intestinal microflora stepwise from the terminal sugar [72,73]. These intestinal microflora include *Prevotella oris *[74], *Eubacterium A-44 *[75], *Bifidobacterium sp*. [73,76], *Bacteroides JY6 *[73], *Fusbacterium K-60 *[73], *Lactobacillus delbrueckii sp*. [76] and *Aspergillus sp*. [76]. Following biodegradation, compound K and protopanaxadiol (PPD) are the major metabolites of PPDs while PPTs are converted to F1 and protopanaxatriol (PPT) (Figure 1).

### Pharmacokinetic and bioavailability of ginsenosides

How intact and transformed ginsenosides are absorbed and transported to the human system remains elusive. Transport of ginsenosides across the intestinal mucosa is energy-dependent and non-saturable [77-79]. The sodium-dependent glucose co-transporter 1 may be involved in this process [80]. The availability of intact ginsenosides and their metabolites from the intestines is extremely low [81-83]. For example, only 3.29% Rg1 and 0.64% Rb1 are detected in rat serum after oral administration of ginsenosides [78,79], confirming the classic studies by Odani *et al*. in 1983 [84,85]. Rg1 levels become undetectable within 24 hours of oral consumption while Rb1 levels remain relatively stable for three days [83].

Experiments to increase the bioavailability of ginsenosides include co-administration of ginsenosides with adrenaline [86], emulsification of ginsenosides into lipid-based formulation [87,88] and suppression of p-glycoprotein efflux system [77]. P-glycoprotein-mediated multidrug resistance is a major obstacle to effective cancer treatments. As ginsenoside Rg3 blocks drug efflux by inhibiting p-glycoprotein activities and reducing membrane fluidity, it is used to assist cancer chemotherapy [28,89,90].

### Ginsenosides are agonists to steroidal receptors

Ginsenosides modulate expressions and functions of receptors such as receptor tyrosine kinases (RTK) [91], serotonin receptors (5-HT) [92], NMDA receptors [93] and nicotinic acetylcholine receptors (AChR) [94]. Direct interactions of ginsenosides with the receptor ligand-binding sites have only been demonstrated in steroid hormone receptors; ginsenosides Rg1 [58,95,96] and Re [97] are functional ligands of the glucocorticoid receptor (GR) while ginsenosides Rh1 and Rb1 are functional ligands of the estrogen receptor (ER), in particular, the ER beta isoform of Rb1 [59,98]. These findings provide an explanation for the aggravation of menopausal symptoms by ginsenosides [99,100] and modulation of the endocrine system in the case of chronic consumption of ginseng [3,4].

Glucocorticoid is a stress hormone to elicit 'fight-or-flight' responses through GR activation. If Rg1 and Re are functional ligands of GR, how is ginseng adaptogenic and antistress? Rg1 and Re may behave as partial agonists to GR. Both Rg1 and Re inhibit the binding of the synthetic glucocorticoid dexamethasone to GR and 100% displacement is possible when ginsenosides are in excess [96,97]. Since Rg1 and Re elicit biological activities that are GR inhibitor RU486 sensitive, indicating these ginsenosides are agonists, but not inhibitors for GR [58,96]. And it is because the steroidal effects of Rg1 and Re are not as prominent as dexamethasone, these ginsenosides are likely to be partial agonist of GR [58,96]. Under physiological conditions, ginsenosides may compensate the insufficient steroidal activities, when the intrinsic ligand is absent or inadequate in the system. On the other hand, ginsenosides can reversibly occupy certain percentage of the steroidal receptor at low affinity to counter the steroidal effects when they co-exist with a large amount of intrinsic ligand.

Moreover, each ginsenoside is able to bind to multiple steroid hormone receptors. In addition to GR, ginsenoside Rg1 acts through ER and elicits cross-talking with insulin-like growth factor-1 receptor (IGF-IR) in neuronal cells [101]. Effects of ginsenoside Re on cardiac myocytes are related to ER alpha isoform, androgen receptor and progesterone receptor [102]. The end-metabolites PD and PT bind and activate both GR and ER in endothelial cells [103]. The multi-target properties of ginsenosides may explain why ginseng has a wide range of beneficial effects.

## Conclusion

As partial agonists to multiple steroidal receptors, ginsenosides are important natural resources to be developed into new modalities, and may replace steroids in the current regimen to lessen undesirable side effects. However, low bioavailablilities of ginsenosides and its metabolites means that most of these compounds do not reach the intended biological system when administered orally. The results of ginsenoside researches will become physiological relevant only when (1) the pure compounds of the ginsenosides is available in large quantities; (2) the ginsenosides are biochemically stabilized to avoid degradation and enhance absorption in the gastrointestinal tract; and/or (3) special delivery methods for the ginsenosides to reach the areas of treatment. Moreover, this review highlighted the necessary of ginsenoside transformation to exert its greatest effects in the mammalian system, thus accelerating this process would help maximizing the remedial effects of ginsenosides. Addressing these two issues in the near future would advance ginseng researches and enhance the possibility for ginseng to be used clinically.

## Abbreviations

5-HT: serotonin receptors; AChR: acetylcholine receptor; ER: estrogen receptor; GR: glucocorticoid receptor; HPLC: high performance liquid chromatography; IGF-IR: insulin-like growth factor-1; PD: panaxadiol; PT: panaxatriol; PPD: 20(S)-protopanaxadiol; PPT: 20(S)-protopanaxatriol; RTK: receptor tyrosine kinases

## Competing interests

The authors declare that they have no competing interests.

## Authors' contributions

KWL and ASTW contributed equally on developing the concept, drafting and editing the manuscript. Both authors read and approved the final version of the manuscript.
